# A case of long-term survival after splenectomy for solitary splenic metastasis from gastric cancer

**DOI:** 10.1186/s12957-020-02024-1

**Published:** 2020-09-19

**Authors:** Ayato Obana, Nobuo Komatsu, Kazuma Aiba, Shinya Nakanishi, Masakazu Abe, Toshiyuki Yamaguchi, Masahiro Hayashi, Hayato Obi, Masamichi Koyama, Shinichi Hashimoto

**Affiliations:** 1 Department of Surgery, Asama Nanroku Komoro Medical Center, Nagano, Japan; 2 Department of General Surgery, Kashiwa Kousei General Hospital, 617, Shikoda, Kashiwa, Chiba, 277-8551 Japan

**Keywords:** Splenic metastasis, Gastric cancer, Splenectomy

## Abstract

**Background:**

Very rarely does a splenic solitary metastasis arise from a gastric carcinoma because splenic metastasis is usually seen in association with widespread visceral metastasis. Splenectomy is considered to be a curative treatment; however, long-term prognosis after splenectomy has scarcely been reported. We report a case of a metachronous and solitary metastasis to the spleen from gastric cancer in which the patient achieved 5-year recurrence-free survival after splenectomy.

**Case presentation:**

An 84-year-old man underwent an open total gastrectomy involving D1+ lymph nodes dissection for gastric cancer located in the cardia (pT3N1M0, pStage IIB). Eighteen months later, a 2-cm solitary hypodense lesion was detected in the spleen by computed tomography (CT). Twenty-three months later, the serum carcinoembryonic antigen (CEA) value elevated to 19.9 ng/ml, and abdominal CT revealed an increase in tumor size to 5 cm. Positron-emission tomography (PET)-CT revealed intense ^18^F-2-deoxy-2-fluoro-glucose (FDG) uptake in the spleen without the involvement of other organs and lymph nodes. We diagnosed him with solitary splenic metastasis from gastric cancer and performed a splenectomy 26 months after the first surgery. Histological examination revealed that the splenic tumor was a moderately differentiated adenocarcinoma, which was very similar to the primary gastric tumor; the lesion was diagnosed as a metastatic tumor from the previous gastric carcinoma. The patient remains healthy to date without recurrence, 5 years after the splenectomy.

**Conclusion:**

We experienced a case of a solitary splenic metastasis from gastric cancer in which 5-year recurrence-free survival was achieved after splenectomy. To determine the surgical indication in patients with splenic metastasis, it is important to differentiate between a solitary lesion or multiple metastasis. Especially, occult metastasis should be excluded by means of several months of follow-up with imaging tests and systemic FDG-PET surveys before splenectomy.

## Background

Splenic metastases from non-hematologic malignancies are quite rare [1] and usually found in association with widespread visceral metastases, according to large retrospective studies on autopsy cases [2, 3]. Solitary splenic metastasis from gastric cancer is even rarer, and only few case reports have been published [4–14]. Those reports concluded that splenectomy could be a potential effective treatment for splenic solitary metastasis. However, all those case reports had a follow-up period of only a few years after the splenectomy, and no long-term prognosis has been reported. We herein report a successful surgical treatment case involving a metachronous solitary splenic metastasis from gastric cancer in which the patient achieved a favorable prognosis with 5-year recurrence-free survival after the splenectomy.

## Case presentation

**Fig. 1:**
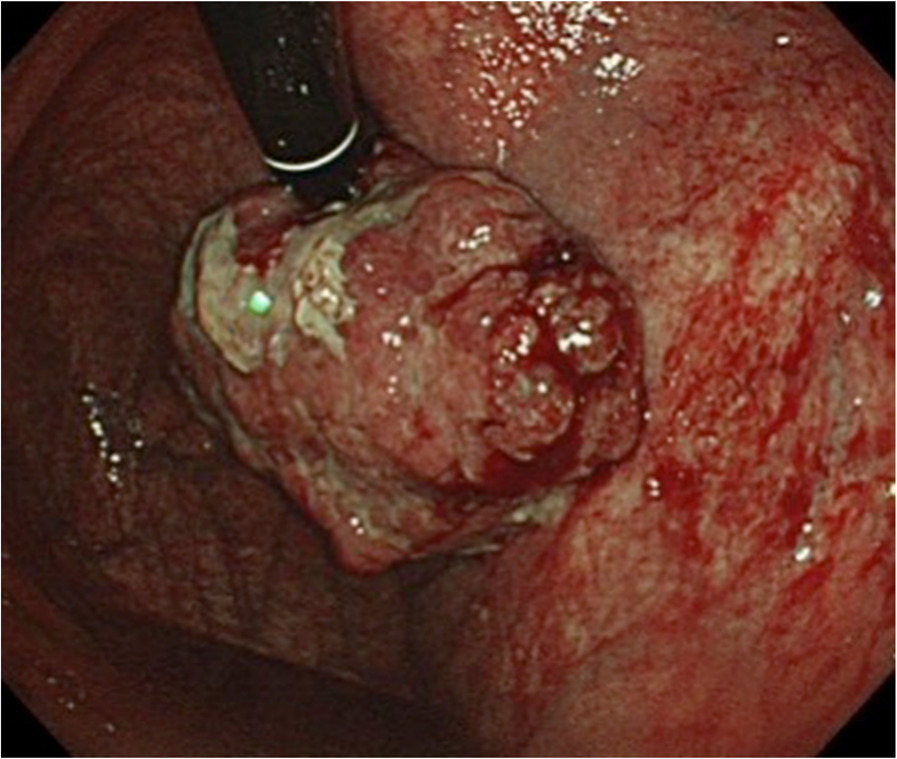
Upper endoscopy detected approximately 5-cm-wide type 1 tumor in the cardia region of the stomach

An 84-year-old Japanese male patient was referred to our hospital for detailed examination for gastric cancer diagnosed at a routine medical check-up. Upper endoscopy was performed and an approximately 5-cm wide type 1 tumor was detected in the cardiac region of the stomach (Fig. 1).

Biopsy showed moderately differentiated adenocarcinoma (tub2). No distant metastatic lesions were identified on enhanced abdominal computer tomography (CT) or chest CT. Laboratory data showed that tumor markers such as carcinoembryonic antigen (CEA) and carbohydrate antigen 19-9 (CA19-9) were within the normal limits. Accordingly, based on the Japanese gastric cancer treatment guideline [15], total gastrectomy with D2 lymph node dissection was indicated. However, considering his older age, total gastrectomy with D1+ regional lymph node dissection was performed. The resected specimen revealed that the tumor was 48 × 28 mm in diameter. Histological examination showed moderately differentiated adenocarcinoma infiltrating the subserosa with metastasis to 1 of 37 regional lymph nodes (a lymph node along the short gastric artery was positive) and slight lymphatic invasion; however, no venous invasion was identified.

According to the Japanese classification of gastric carcinoma 3rd English edition [16], the patient was diagnosed with pT3N1M0, ly1, v0, and pStage IIB. Postoperative course was uneventful, and the patient was discharged on postoperative day 17. Adjuvant chemotherapy with oral TS-1 was recommended, according to the Japanese gastric cancer treatment guideline [15]. However, considering his older age, this regimen was not performed and the patient was followed-up in the outpatient clinic.

The patient was assessed according to the Japanese gastric cancer treatment guideline, which was comprised of routine physical examinations, measurements of serum tumor markers such as CEA and CA 19-9 (every three months during the 5 years after the surgery), thoracoabdominal computed tomography (every 6 months during the first 3 years after the surgery and once every 12 months from the fourth year onward), and upper endoscopy (1, 3, and 5 years after the surgery).

**Fig. 2:**
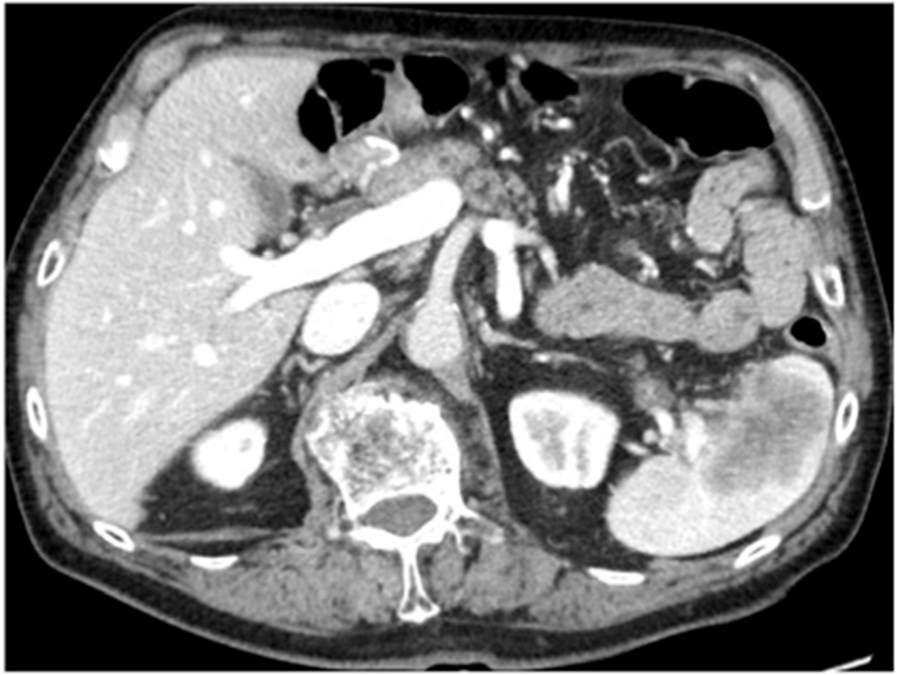
A hypodense lesion (diameter 5 cm) was identified on enhanced abdominal CT 23 months post-first surgery. CT, computed tomography

Eighteen months later, a 2-cm solitary hypodense lesion was detected in the spleen on CT, but serum tumor markers remained within the normal limits. Twenty-three months later, serum CEA elevated to 19.9, and an abdominal CT revealed that the splenic lesion increased in size to about 5 cm (Fig. 2).

**Fig. 3:**
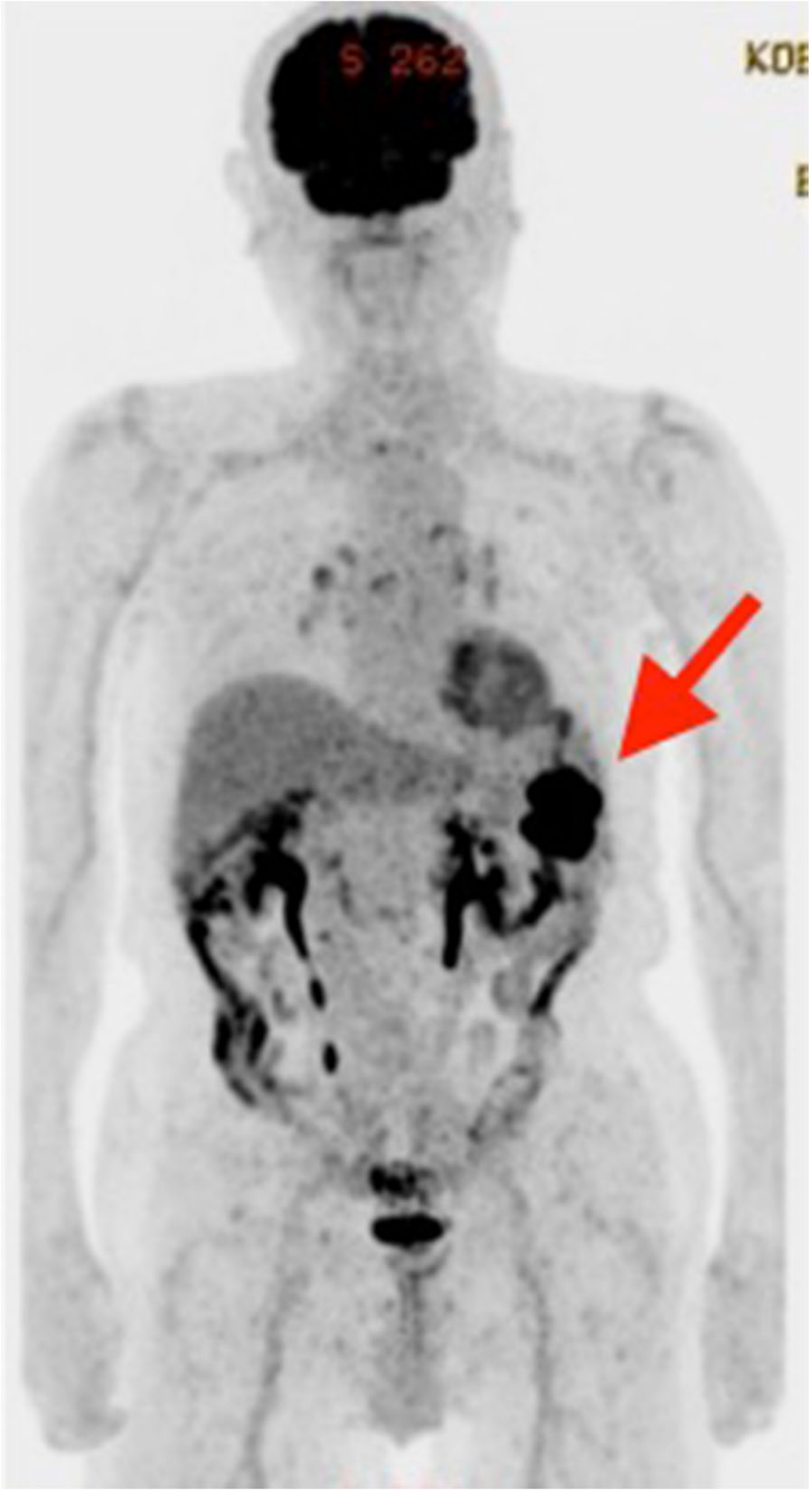
FDG-PET–CT showing abnormal FDG accumulation in the spleen (red arrow). FDG, ^18^F-2-deoxy-2-fluoro-glucose, PET–CT, positron emission tomography–computed tomography

Splenic metastasis was suspected, and ^18^F-2-deoxy-2-fluoro-glucose (FDG) positron emission tomography–CT (PET/CT) was scheduled to identify other metastatic sites besides the spleen. The PET-CT revealed intense FDG uptake in the spleen without involvement of other organs (Fig. 3). Upper endoscopy and colonoscopy were also performed, and no abnormalities were identified.

We diagnosed the patient with solitary splenic metastatic tumor from gastric cancer and thought the splenectomy could be an effective treatment to eliminate the tumor even though his age was 86 at that time. Therefore, the splenectomy was performed 26 months after the first surgery.

**Fig. 4:**
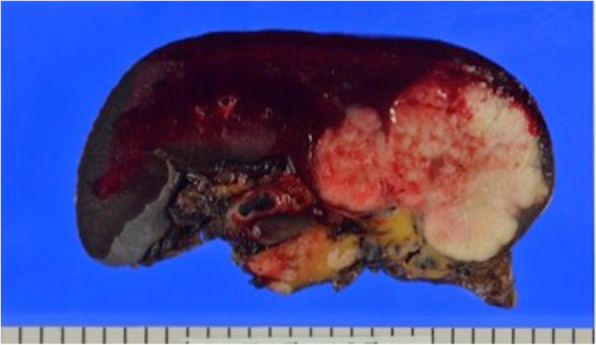
The resected specimen has a smooth surface and contains a well-circumscribed solid tumor

Resected specimen showed a well-circumscribed, white, 57 × 51 mm in size, solid tumor located in the splenic parenchyma on cross-section. The tumor was demarcated from the splenic parenchyma without any capsule invasion (Fig. 4). Histological examination revealed that the splenic tumor was a moderately differentiated adenocarcinoma, which was very similar to the primary gastric cancer. The immunohistochemistry result of both the gastric cancer and the splenic tumor showed positive for cytokeratin 7, CEA, and negative for cytokeratin 20, p53. These histological and immunochemical findings were consistent with primary gastric cancer and splenic tumor. Therefore, the lesion was diagnosed as metastasis from the previous gastric carcinoma.

The postoperative course was uneventful, and the patient was discharged on postoperative day 21 after the splenectomy. No chemotherapy was administered considering his age, and he was followed-up in the outpatient clinic. The patient remains well to date without recurrence and achieved 5 years of recurrence-free survival after splenectomy for solitary splenic metastatic lesion from gastric cancer.

## Discussion and conclusions

This case highlighted two important issues. Solitary splenic metastasis can occur after the gastrectomy for gastric cancer. Splenectomy is a potentially effective treatment for solitary splenic metastasis when occult metastasis is ruled out by means of several months of follow-up with imaging tests and PET/CT.

The first issue is that a solitary splenic metastasis can occur after the gastrectomy for gastric cancer. Except for leukemia and malignant lymphoma, splenic metastasis is infrequent. Among 4407 autopsy cases associated with malignant metastasis, splenic metastasis was found in 312 cases (7.1%), and 22 cases showed splenic metastasis among 536 cases of gastric cancer (4.1%) [17]. Splenic metastasis from gastric cancer usually presents as one of the multi-organ involvements [2, 3], and there are only few case reports about solitary splenic metastasis from gastric cancer. Actually, there is no definitive reason despite several hypotheses for this [1, 2]. First, the spleen has a poorly developed lymphoid system especially in afferent lymphatic vessels. It is rare for a tumor cell to metastasize via the lymphatic route. Second, the splenic artery arises at a sharp angle from the celiac artery. It is very difficult for tumor cells to reach the spleen. Third, the spleen has an immunological antitumor effect and rhythmically constricts to squeeze out tumor cells. When solitary splenic metastasis is suspected, it is expected to be accompanied with multi-organ occult tumor metastases. To differentiate solitary or widespread visceral metastasis, the wait-and-see approach is suggested; follow-up with the suspected patient performing image tests for a couple of months and wait for the growth of occult tumor metastasis [5, 13, 14]. However, there is no consensus about the follow-up interval of the wait-and-see approach before splenectomy. In our case, we strongly suspected the patient to have metastases to sites other than the spleen and thus, performed this wait-and-see approach for almost 6 months before a PET/CT assessment. The PET/CT showed abnormal FDP accumulation in the spleen, and we diagnosed solitary splenic metastasis. With the PET/CT after the wait-and-see approach, we differentiated solitary splenic metastasis from widespread visceral metastasis.

The second issue is that splenectomy is a potentially effective treatment for patients with solitary splenic metastasis when multi-organ occult metastasis is ruled out by means of several months of follow-up with imaging tests and PET/CT. There are a reported 12 cases of solitary splenic metastasis from gastric cancer among publications between 2000 and 2020 searched in PubMed [4–14], and our case is the 13th one achieving the longest recurrence-free survival. Both clinical and pathological details of the previous case reports and present case are described in Table 1. In all cases, splenectomy was performed, but there is no report describing the long-term follow-up course after splenectomy. One case report followed up with the patient for 40 months after splenectomy, and the patient died of tumor recurrence in the liver and peritoneum [4]. In this case, we assumed this patient might have occult tumor metastasis in areas other than the spleen because the tumor metastasized via the splenic artery. There are three routes through which gastric cancer cells can metastasize to the spleen [18]: splenic vein, splenic artery, and lymphatic vessels. In the splenic vein, blood flows away from the spleen unless the patient has portal hypertension disease, so it is uncommon for tumor cells to reach the spleen against blood flow through the splenic vein. Via the splenic artery, tumor cells have to pass through the systemic circulation to reach the spleen, and in such cases, splenic metastasis would be accompanied by multi-organ metastasis. As for the lymphatic route, it is true that the spleen has poorly developed afferent lymphatic vessels. However, gastric cancer cells can metastasize directly to the spleen, and solitary splenic metastasis from gastric cancer can occur, theoretically. In our case, the gastric cancer was located in the cardia of the stomach near the spleen, and one lymph node near the short gastric artery was positive for metastasis. In addition, pathological findings showed lymphatic invasion but no vascular invasion. Accordingly, we assumed that the gastric cancer metastasized directly to the spleen through the lymphatic route. Therefore, even though the patient did not have adjuvant chemotherapy after the gastrectomy and splenectomy considering his older age, there were no widespread multi-organ metastases and the patient achieved a 5-year recurrence-free survival after the splenectomy.

**Table 1:** Summary of patients with solitary splenic metastasis from gastric cancer treated by splenectomy

				Gastric cancer							Splenic metastasis			Prognosis	
Case	Author	Age	Sex	Location	Size (mm)	TMN	Histology	Procedure	Adjuvant chemo	DFI	Size (mm)	Suggested route	Operation	Follow-up	Outcome
2000	Opocher	76	M	L	Unknown	T2N0M0	Tub	DG	N/A	57 months	80	Hematogenous	Splenectomy	13 months	RFS
		66	M	Unknown	Unknown	N2N1M0	Por	TG	N/A	36 months	40	Hematogenous	Splenectomy	14 months	RFS
2002	Yamanouchi	69	M	L	Unknown	T3N2N0	Tub	DG	N/A	48 months	45 × 40	Hematogenous?	Splenectomy	40 months	Dead
2009	Sunitsch	80	F	L,R	Unknown	T2N0M0	Tub	TG	N/A	37 months	150	N/A	Splenectomy	N/A	N/A
2010	Lu	59	M	U	70	TXNXM1	Hepatoid AC	TG + S	N/A	Same	40	N/A	Same (synchronous)	18 months	Alive
2010	Kawasaki	76	M	U	20	T1N1M0	Pap	EMR and PG	N/A	12 months	65	Hematogenous?	Chemo+splenectomy	24 months	RFS
2011	Deng	49	M	M	60	T3N2M1	Tub	DG	Performed	60 months	140×100×70	Hematogenous	Splenectomy	9 months	RFS
2013	Kamaleshwaran	55	M	U	Unknown	Unknown	Unknown	PG	N/A	12 months	Unknown	N/A	Splenectomy	N/A	N/A
2013	Zhu	62	M	M,L	90×80	T3N2M0	Por	TG	Performed	12 months	45×35, 30 × 20	N/A	Splenectomy	8 months	RFS
2015	Santos	71	M	Unknown	Unknown	T3N0M0	Unknown	TG	N/A	72 mo	25 × 15 × 10, 15, 10	Hematogenous	Splenectomy	7 months	RFS
2017	Yoshizawa	60	M	M	25 × 22	T1N2M0	Tub, Por	DG	Performed	12 months	20 × 18	Lymphatic	Splenectomy	18 months	RFS
2017	Namikawa	75	M	U	22	T1N0M0	Tub	ESD and TG	N/A	28 months	55 × 45	N/A	Splenectomy	2 months	RFS
2020	Obana	84	M	U	48 × 28	T3N1M0	Tub	TG	N/A	15 months	57 × 51	Lymphatic	Splenectomy	60 months	RFS

*DFI* Disease-free interval, *U* The upper third of stomach, *M* Middle third of stomach, *L* Lower third of stomach, *R* Residual stomach, *Tub* Tubular adenocarcinoma

*Por* Poorly differentiated adenocarcinoma, *Pap* Papillary adenocarcinoma, *AC* Adenocarcinoma, *N/A* Not available, *DG* Distal gatrectoy

*TG* Total gastrectomy, *PG* Proximal gastrectomy, *S* Splenectomy, *RFS* Relapse-free survival, *ESD* Ensoscopic submucosal dissection

There were 12 case reports of solitary splenic metastasis from gastric cancer before us. In those case reports, splenectomy was performed but follow-up periods were very short after the splenectomy

Though it is a very rare case, splenic metastasis can occur not only from gastric cancer but also from other non-hematopoietic malignancies such as colorectal cancer, ovarian cancer, and lung cancer [19]. Regardless of the primary lesion, splenic metastasis is usually associated with multi-visceral organ metastases. When we suspected a solitary splenic metastasis, we performed a wait-and-see approach first and then performed PET/CT evaluation to rule out other organ metastases before splenectomy.

Solitary splenic metastasis can occur after gastrectomy for gastric cancer. When a solitary splenic metastasis is suspected, the patient should be evaluated with imaging tests for several months to rule out other organ metastasis with PET/CT before splenectomy.

## Data Availability

Data sharing is not applicable to this article as no datasets were generated or analyzed during the current study.

## Abbreviations

CA19-9: Carbohydrate antigen 19-9
CEA: Carcinoembryonic antigen
CT: Computed tomography
FDG: ^18^F-2-deoxy-2-fluoro-glucose
PET: Positron-emission tomography
